# Genome-wide association mapping of spot blotch resistance in wheat association mapping initiative (WAMI) panel of spring wheat (*Triticum aestivum *L.)

**DOI:** 10.1371/journal.pone.0208196

**Published:** 2018-12-17

**Authors:** Ram Narayan Ahirwar, Vinod Kumar Mishra, Ramesh Chand, Neeraj Budhlakoti, Dwijesh Chandra Mishra, Sundeep Kumar, Shweta Singh, Arun Kumar Joshi

**Affiliations:** 1 Department of Genetics and Plant Breeding, Institute of Agricultural Sciences, Banaras Hindu University, Varanasi, India; 2 Department of Mycology and Plant Pathology, Institute of Agricultural Sciences, Banaras Hindu University, Varanasi, India; 3 ICAR- Indian Agricultural Statistics Research Institute, New Delhi, India; 4 ICAR- National Bureau of Plant Genetic Resources, New Delhi, India; 5 International Maize and Wheat Improvement Center (CIMMYT), DPS Marg, New Delhi, India; 6 Borlaug Institute for South Asia (BISA), DPS Marg, New Delhi, India; Mahatma Phule Krishi Vidyapeeth College of Agriculture, INDIA

## Abstract

Spot blotch (SB) caused by *Bipolaris sorokiniana*, is one of the most important diseases of wheat in the eastern part of south Asia causing considerable yield loss to the wheat crop. There is an urgent need to identify genetic loci closely associated with resistance to this pathogen for developing resistant cultivars. Hence, genomic regions responsible for SB resistance were searched using a wheat association mapping initiative (WAMI) panel involving 287 spring wheat genotypes of different origin. Genome-wide association mapping (GWAM) was performed using single nucleotide polymorphism (SNP) markers from a custom 90 K wheat SNP array. A mixed linear model (MLM) was used for assessing the association of SNP markers with spot blotch resistance in three consecutive years. Three traits were measured: incubation period, lesion number and area under the disease progress curve (AUDPC). Significant SNP markers were found linked to five, six and four quantitative trait loci (QTLs) for incubation period, lesion number and AUDPC respectively. They were detected on 11 different chromosomes: 1A, 1B, 1D, 4A, 5A, 5B, 6A, 6B, 6D, 7A, 7B with marker R^2^ range of 0.083 to 0.11. The greatest number of significant SNP-markers was found for lesion number and AUDPC on chromosome 6B and 5B, respectively, representing a better coverage of B-genome by SNPs. On the other hand, the most significant and largest SNP markers for incubation period were detected on 6A and 4A chromosomes indicating that this trait is associated with the A-genome of wheat. Although, QTLs for spot blotch resistance have been reported in wheat on these same chromosomes, the association of incubation period and lesion number with SB resistance has not been reported in previous studies. The panel exhibits considerable variation for SB resistance and also provides a good scope of marker-assisted selection using the identified SNP markers linked to resistant QTLs.

## Introduction

Wheat (*Triticum aestivum* L.), one of the most widely adapted cereal crops [1], provides around one-fifth of the total calories to the human population. In addition, it also provides 20% of the protein to more than 4.5 billion people in 94 developing countries [2]. In south Asia, wheat is cultivated in over 40 million hectare area, out of which around 25% falls under Eastern Gangetic Plains (EGP) which contributes only 16% to the total wheat production. The demand of wheat in this densely populated region can only be met if annual production is increased by 1.5–2.0% [3, 4]. The EGP of south Asia is characterized as the mega environment 5A due to prevalence of high temperature and humidity during the wheat growing season [5, 6].In this region, wheat production is significantly impacted due to spot blotch disease [6, 7, 8] which may cause yield loss up to 30% [9]. The causal organism of spot blotch (SB) is *Bipolaris sorokiniana*, which is an anamorph (teleomorph *Cochliobolus sativus*).

Reduction in disease progress is contributed by few components of resistance i.e., latent period and lesion size [10] and epidemic development can be slow down by using genotypes with longer latent period and fewer lesions [11]. Hence molecular mapping for different components of resistance in plant populations to determine robust markers and their subsequent use is imperative for varietal improvement programs through an effective use of appropriate genetic resources [12, 13].Due to various limitations of the conventional bi-parental linkage mapping, there has been a greater preference for linkage disequilibrium (LD) based association mapping (AM) [6]. In AM, a collection of variable lines is scanned to understand marker-trait associations using linkage disequilibrium (LD)which is non-equilibrium association between different alleles at various loci [14] and varies across wheat chromosomes [12, 15]. Although wheat has a large homeologous genome that shows weak marker coverage compared to other cereal crops [13], AM can identify superior alleles with detailed marker data in large populations which can be utilized immediately in breeding [16].

Due to significant yield losses caused by spot blotch in EGP, a considerable effort has been invested by wheat breeders to develop spot blotch resistant cultivars in this part of South Asia. To continue this effort further, this study was undertaken to identify novel marker-trait associations and to detect the loci conferring spot blotch resistance through GWAM in a WAMI Panel.

## Materials and methods

### Experimental material

The experimental material comprised of 287 genetically diverse, elite spring wheat lines of the wheat association mapping initiative (WAMI) population assembled by CIMMYT in 2009 was used [17].

### Experimental design and trait evaluation

The WAMI population was evaluated for spot blotch in three consecutive crop seasons during 2012–13, 2013–14 and 2014–15 at the Agricultural Research Farm of Banaras Hindu University (BHU), Varanasi, India. The experiment was conducted using Alfa lattice design with sowing done between 1^st^ to 10^th^ December in the three years. Two replications were planted and each genotype was sown in two rows of two meters maintaining row-to-row and plant-to-plant distance of 20 cm and 5 cm respectively. The lines were allocated randomly to each replication using the Fisher and Yates Random Table [18]. Fertilizer per hectare was applied as 120 kg N: 60 kg P_2_O_5_: 40 kg K_2_O. The full amount of K_2_O and P_2_O_5_ were applied at sowing whereas nitrogen was applied in three split doses, half at sowing, one fourth at first irrigation (21 days after sowing-DAS) and the remaining one fourth at second irrigation (45 DAS). Data were recorded on three traits: incubation period (days), lesion number and spot blotch AUDPC.

### Inoculation of pathogen

For creating artificial epiphytotic, a pure culture of *Bipolaris sorokiniana* (NABM MAT1; NCBIJN128877, BHU, Varanasi, India) known to be highly aggressive was used [19]. The isolate was obtained from the Department of Plant Pathology and Mycology, BHU and multiplied on sorghum grain. An adjusted spore suspension (10^−4^ spores per ml) in water was applied in the evening and irrigated immediately after inoculation [20]. Frequent irrigations were provided to ensure environmental conditions conducive to spot blotch development in the field.

### Recording for incubation period, lesion number and disease score

Incubation period (days) was recorded from the inoculation to first appearance of visible symptoms on five randomly tagged plants in each plot [10]. For number of lesions, five leaves from each plot were selected randomly. Total numbers of lesions present on flag leaves were counted. The leaf was divided into four parts with a marker pen and the number of lesions on each part was counted. Total number of spots of each part were added and recorded as lesions per leaf [21]. For AUDPC, disease severity (%) was recorded in three different growth stages (GS), GS 63 (beginning of anthesis to half complete), GS 69 (anthesis complete) and GS 77 (late milking). Disease severity was assessed by the formula (D_1_/9× D_2_/9) × 100 using the double digit scale (DD, 00–99) [7]. The first digit (D_1_) refers to vertical disease progress on the plant whereas the second digit (D_2_) was the disease severity score in the affected leaves. Thus, disease severity was used to estimate the AUDPC by following formula [22].

$$AUDPC=\overset{n-1}{\underset{i=0}{\sum }}\left[\left\{\left(Y_{i}+Y_{i+1}\right)/2\right\}\times \left(t_{i+1}-t_{i}\right)\right]$$

Where, *Y*_*i*_ = disease severity at time *t*_*i*_, (*t*_*i+1*_−*t*_*i*_) = Time (days) between two disease scores, *n* = number of dates at which spot blotch was recorded.

### DNA extraction and SNP Genotyping

The DNA was extracted from fresh leaves of each line following the CTAB procedure [23] and genotyped at CIMMYT, Mexico using the Illumina iSelect beadchip assay [17]for wheat having 26814 SNPs. To avoid monomorphic and low-quality SNPs, markers were removed with a minor allele frequency (MAF) <0.05. Nearly 21132polymorphic SNPs were selected and used for AM.

### Phenotypic and STRUCTURE analysis

Analysis of variance (ANOVA) was completed using SAS 9.4 to determine genotypic, year and genotypic × year variances among the traits measured over the years. Population structure (Q) was analyzed for filtered marker panel through a clustering approach based on the admixture model implemented in STRUCTURE [24]. Ten iterations of STRUCTURE runs were employed for each subpopulations *K* that ranged from 2 to 10 with additional parameters of 10,000 burn-in length and 10,000 Markov chain Monte Carlo (MCMC) iterations. Clusters with the greatest likelihood of occurrence were identified through the *ΔK* method [25]. The web-based tool for population structure analysis “Structure Harvester” [26] was done which maximizes ΔK and thus examines the likelihood distribution of K. Further, fixation index (Fst) for subpopulations was estimated by outputs generated from various STRUCTURE runs. Population Matrix *Q* was also obtained for further analysis. TASSEL 5.0 was used to calculate population Kinship matrix, which is based on scaled IBS (identity by state) method that uses marker data which has passed quality filtering.

### Genome-wide association analysis

Marker–trait associations (MTAs) were detected by Mixed Linear Model (MLM) in TASSEL 5.0 (http://www.maizegenetics.net/) [27]. The general equation used for MLM analysis was as follows:

y=Xβ+Qv+Zu+e

Where, *y* = phenotype vector, *β* = vector of marker fixed effects; *v* = vector of fixed effects; *u* = vector of random effects (the kinship matrix); *e* = vector of residuals; *X* = genotype of a marker; *Q* = Kinship Matrix (obtained using STRUCTURE software) and *Z* = Identity matrix which considers the familial relatedness between accessions.

The *Q* matrix was used as a covariate to improve the precision of results by avoiding false positives. The efficient mixed model analysis [28] was used to save computing time, while other MLM parameters were kept as default. MLM, also called *Q* + *K* model, takes into account the population structure as well as familial relatedness. Significant marker-trait associations were identified based on P-value and R^2^ used for estimating the magnitude of the QTL effects. Significant marker-trait associations were selected on basis of P-values ≤ 1e-06. In addition, pair-wise LD for pairs of markers was calculated using TASSEL 5.0 considering observed and expected allele frequencies of the markers.

## Results

The results of the analysis of variance (ANOVA) are presented in Table 1. The traits-incubation period, lesion number and AUDPC revealed significant (P<0.01) differences among genotype, years and genotype × year (Table 1).

**Table 1:** Analysis of variance for incubation period (IP) lesion number (LN) and Area under disease progress curve (AUDPC).

Source	d.f.	IP		LN		AUDPC	
		Mean square	F value	Mean square	F value	Mean square	F value
**Genotype**	287	14.69*	17.55	1732.66*	42.64	102967.85*	30.95
**Year**	2	190.83*	227.98	787826.96*	19386.40	553399.58*	166.35
**Replication**	1	7.13*	8.52	114.66	2.82	10928.33	3.28
**Genotype×year**	574	2.19*	2.62	1076.66*	26.49	18934.13*	5.69

*Significant at the p-value (*P*<0.01) probability level; d.f., degrees of freedom.

**Fig 1:**
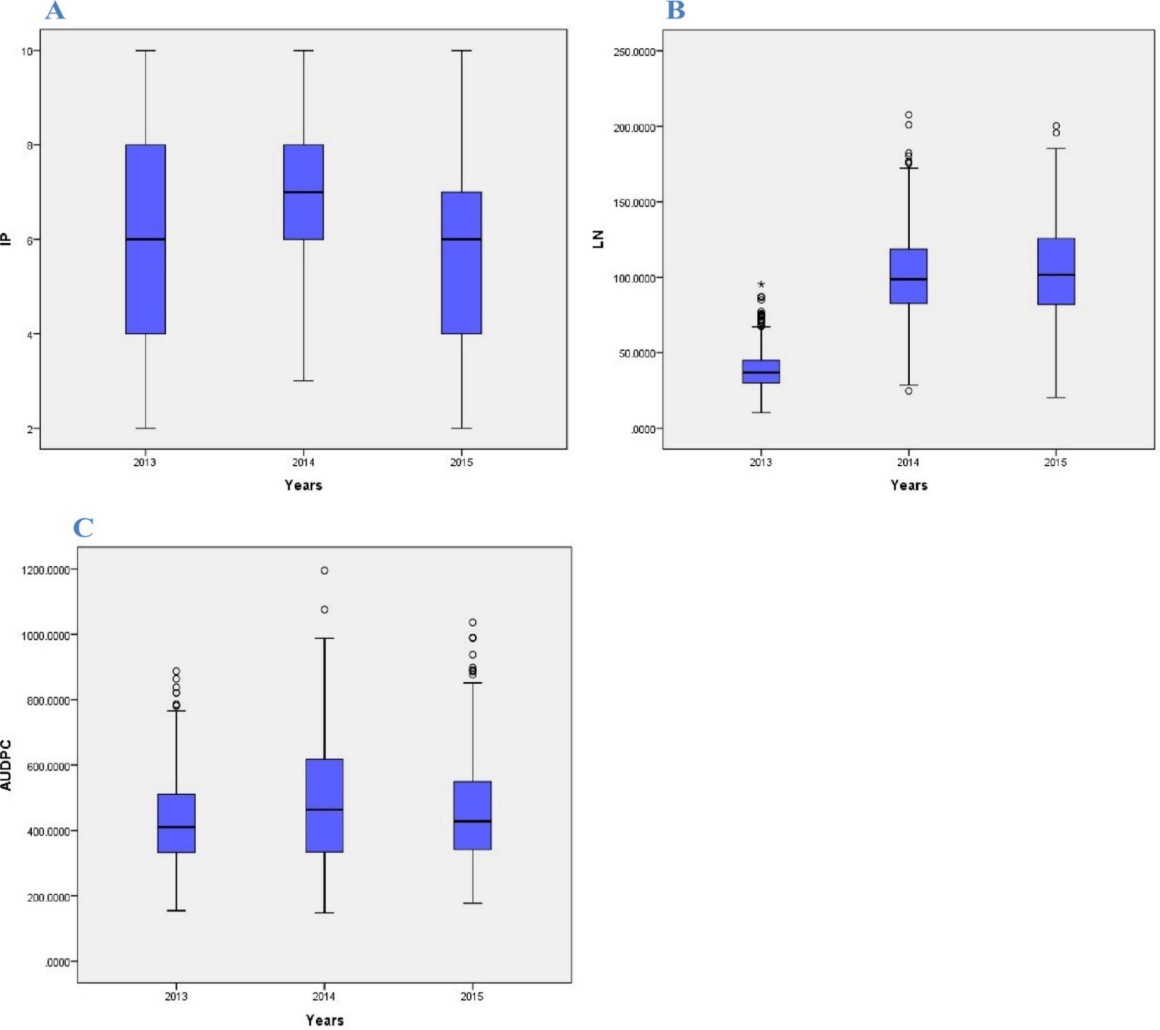
Boxplots of the three different traits showing variation for three different years in 2012–13, 2013–14 and 2014–15. (A) IP (Incubation Period) (B) LN (Lesion Number) (C) AUDPC (Area under disease progressive curve).

**Fig 2:**
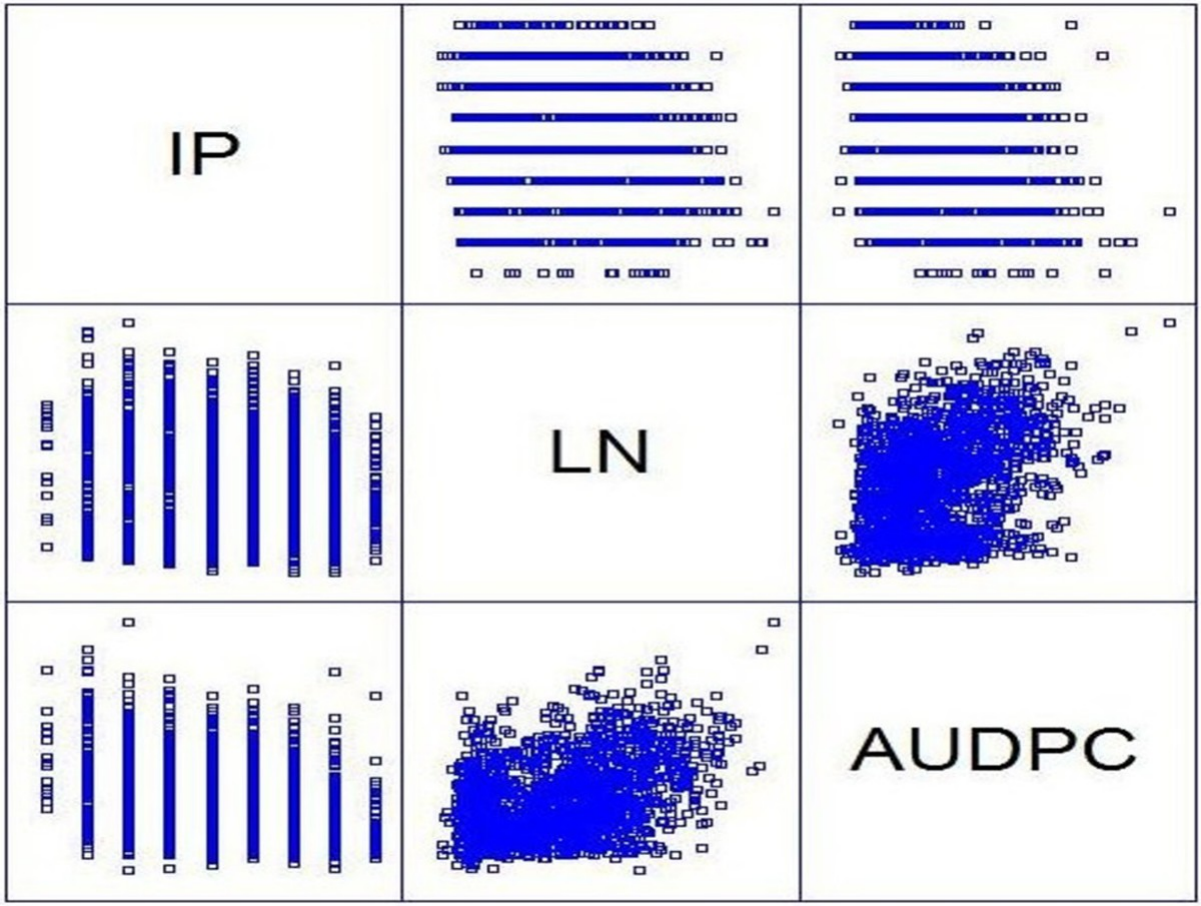
Graphical representation of phenotype correlation among three traits (IP, LN & AUDPC) in WAMI panel.

Distribution of phenotype under study has been generated which shows how performance of phenotype varies over the years (Fig 1A, 1B and 1C). More over to get the better description of phenotypes, correlation among traits under study has also been investigated (Fig 2).

### Population structure and marker distribution

**Fig 3:**
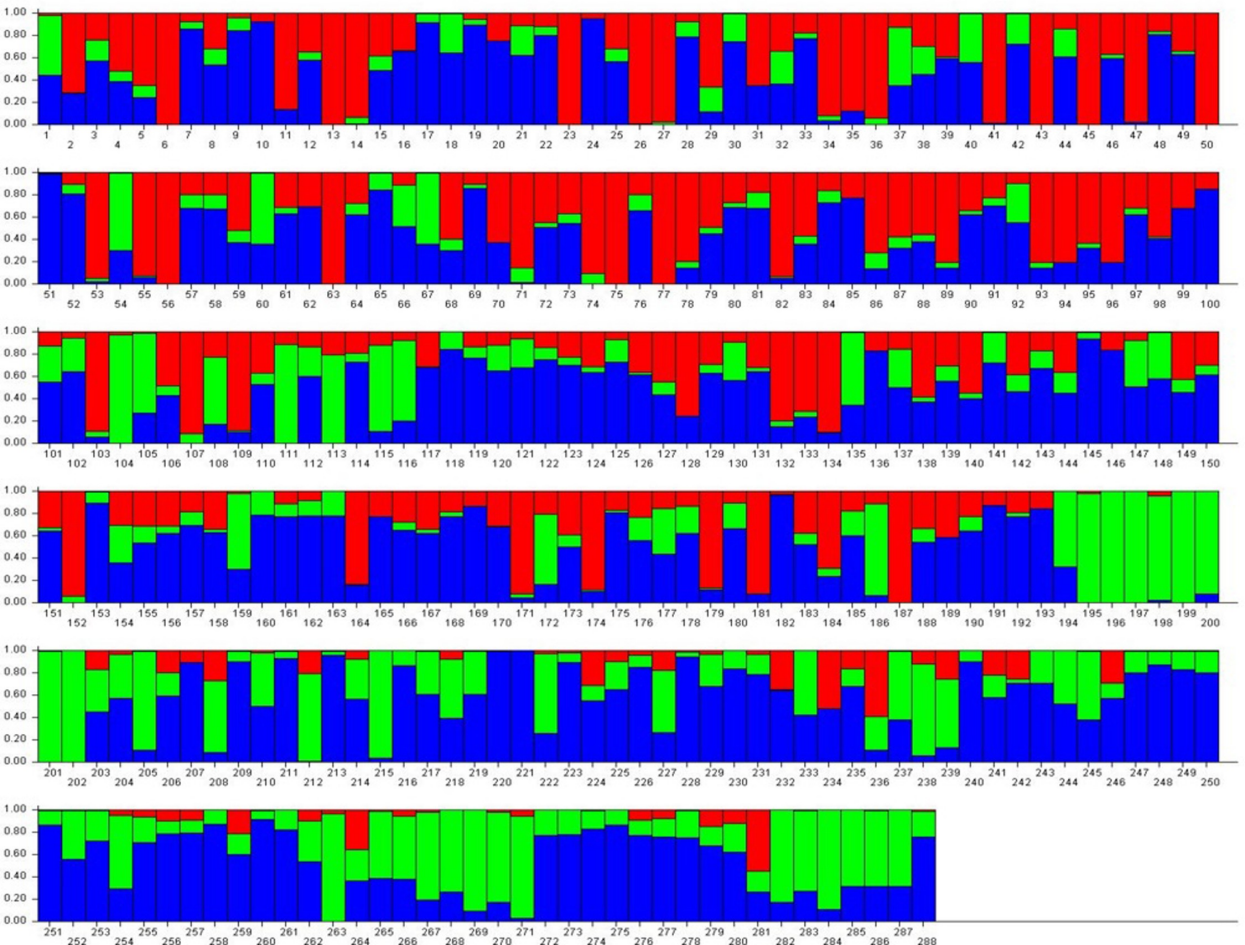
Bar graphs for three subpopulations (estimated following the approach of Evanno, 2005) are indicated by different colors. The vertical coordinates of each subpopulation indicates the membership coefficient for each individual. The horizontal axis shows genotypes under study. In each sub population, each vertical bar represents one genotype.

**Fig 4:**
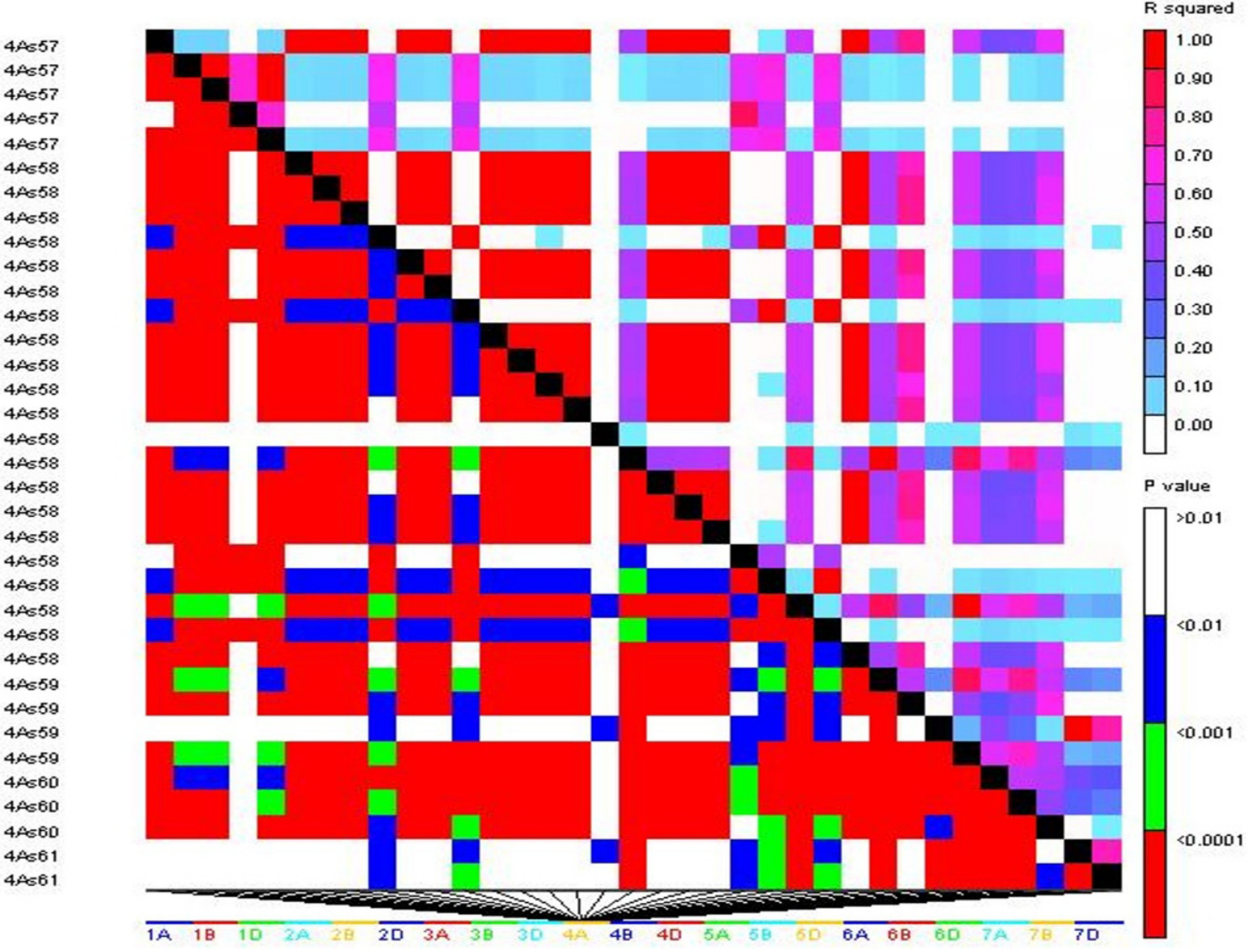
Linkage disequilibrium plots of significant SNP markers associated with spot blotch AUDPC. R square (R^2^) and P-value of pair-wise analyses are indicated by color in the right-side bars.

Optimal number of cluster i.e. K was determined to be 3 based on delta K approach implemented in STRUCTURE [24]. A bar graph representing population structure is also generated (Fig 3). A total 21132 marker which has passed quality filtering were used for the purpose of mapping. Linkage Disequilibrium (LD) plot based on association among markers has also been generated (Fig 4). It is clear from the figure that most of the markers are tightly linked as large chunks of red blocks observed below the diagonal of figure. It suggests that there has been limited scope of recombination between the markers, which facilitates association mapping of all three traits and minimum number of markers required to effectively cover the entire genome. More detailed distribution of marker over chromosome is presented in Table 2.

**Table 2:** Distribution of 21,132 SNPs in 21chromosomes identified in 287 wheat genotypes.

Chr.	Size (Mb)	No. of SNP	Average number of SNPs per Mb	Chr. LD
1A	594.1	1346	2	0.289
1B	689.85	1953	3	0.325
1D	495.45	540	1	0.399
2A	780.8	1176	2	0.305
2B	801.26	2064	3	0.306
2D	651.85	679	1	0.417
3A	750.84	970	1	0.207
3B	830.83	1403	2	0.252
3D	615.55	284	1	0.159
4A	744.59	925	1	0.249
4B	673.62	589	1	0.206
4D	509.86	74	1	0.111
5A	709.77	1151	2	0.275
5B	713.15	1995	3	0.303
5D	566.08	197	1	0.123
6A	618.08	1308	2	0.287
6B	720.99	1509	2	0.298
6D	473.59	199	1	0.115
7A	736.71	1281	2	0.184
7B	750.62	1298	2	0.247
7D	638.69	191	1	0.140

### Genome-wide Marker-traits association with SNP-markers

**Fig 5:**
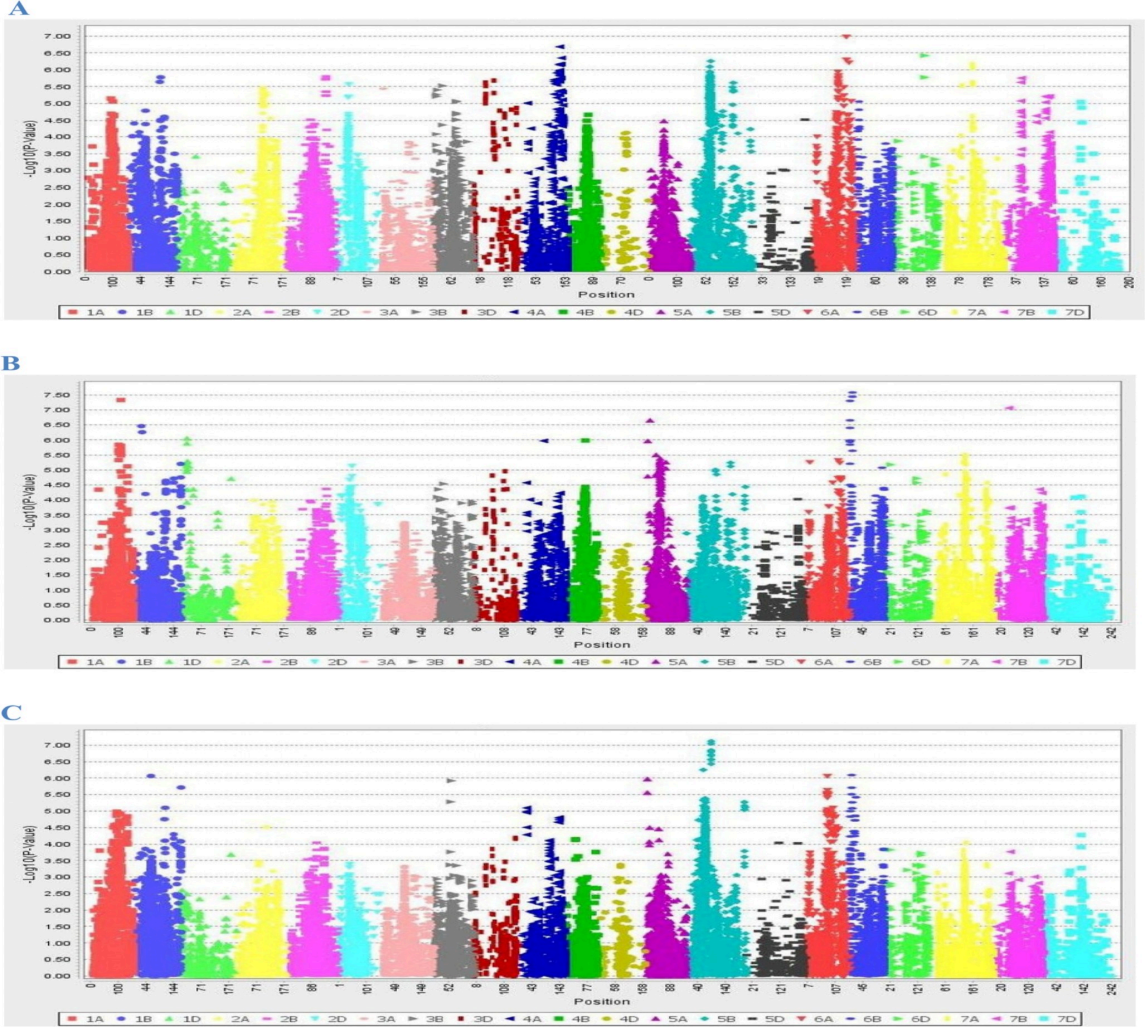
Manhattan plots for the GWAM scan on different traits with p value threshold (-log10 (*P*-value)>6.0). (A) Incubation period, (B) Lesion number, (C) AUDPC.

In the present study, several markers associated with attributes like incubation period, lesion number and AUDPC were identified. The number of SNPs found significant at P-value threshold 1e-06 in the marker-trait association for the three traits, incubation period, lesion number and AUDPC were 13, 8 and 14, respectively (Table 3 and Fig 5A, 5B and 5C). A total of 35 SNPs markers were detected on eleven chromosomes (1A, 1B, 1D, 4A, 5A, 5B, 6A, 6B, 6D, 7A and 7B) with coefficient of determination (R^2^) ranging between 0.083 and 0.11 (Table 3).

**Table 3:** Significant single nucleotide polymorphism (SNP) markers associated with incubation period (IP), lesion number (LN) and Area under disease progress curve (AUDPC) in 287 spring wheat lines in the WAMI panel.

Trait	Markers	Chromosome	Position (cM)	*p*-value	Marker R^2^
**IP**	BobWhite_c20306_111	4A	137	2.08E-07	0.093
	BobWhite_c11327_185	4A	137	6.81E-07	0.085
	BobWhite_c20322_153	4A	137	7.17E-07	0.084
	BobWhite_c17524_242	4A	144	4.47E-07	0.089
	Tdurum_contig81450_90	4A	142	6.81E-07	0.087
	Excalibur_rep_c69981_75	6A	119	1.06E-07	0.098
	wsnp_Ra_c2270_4383252	6A	119	5.07E-07	0.089
	BS00004466_51	6A	128	6.15E-07	0.089
	Ku_c15750_761	7A	126	6.97E-07	0.086
	TA002294-0887	7A	126	8.96E-07	0.086
	Kukri_c23752_659	5B	71	5.55E-07	0.089
	Excalibur_c96334_93	5B	68	8.10E-07	0.093
	Tdurum_contig75700_411	6D	117	3.78E-07	0.091
**LN**	RAC875_c32379_216	1A	110	4.68E-08	0.110
	BobWhite_c23992_300	5A	16	2.23E-07	0.102
	BobWhite_c17559_105	1B	28	3.45E-07	0.096
	wsnp_Ex_c13421_21142520	1B	31	5.51E-07	0.090
	RAC875_c68978_126	6B	9	2.62E-08	0.108
	BS00092845_51	6B	9	3.63E-08	0.104
	wsnp_Ex_c2103_3947695	7B	50	8.65E-08	0.107
	Excalibur_rep_c105429_528	1D	21	8.92E-07	0.088
**AUDPC**	BobWhite_c35961_80	6A	79	8.71E-07	0.083
	BobWhite_c3661_88	1B	64	8.63E-07	0.083
	tplb0027f13_1493	5B	90	7.64E-08	0.101
	wsnp_Ku_c40334_48581010	5B	90	8.99E-08	0.099
	BobWhite_c48435_165	5B	90	1.44E-07	0.096
	Tdurum_contig25513_195	5B	90	1.48E-07	0.095
	Tdurum_contig12066_126	5B	90	1.60E-07	0.093
	Tdurum_contig25513_123	5B	90	1.95E-07	0.093
	tplb0027f13_1346	5B	90	2.08E-07	0.093
	BS00010590_51	5B	90	2.72E-07	0.090
	Tdurum_contig12066_247	5B	90	2.76E-07	0.090
	IACX9261	5B	90	3.71E-07	0.090
	BS00009311_51	5B	62	5.62E-07	0.090
	Excalibur_c96134_182	6B	5	8.09E-07	0.087

For incubation period, 13 SNP markers were detected on five chromosomes in variable numbers; 5 on 4A, 3 on 6A, 2 on 7A, 2 on 5B and 1 on 6D (Table 3)whereas no MTA was found on rest of the chromosomes (Fig 5A). These markers were significant at P values less than 1e-06 and R^2^ for significant markers ranged from 0.084 to 0.098 (Table 3). A prominent QTL was identified for incubation period on 6A Chromosome at 199 cM. Out of five markers detected on 4A chromosome, three markers (BobWhite_c20306_111, BobWhite_c11327_185 and BobWhite_c20322_153) were mapped at 0 cM and the other two (BobWhite_c17524_242 and Tdurum_contig81450_90) at 2 cM away. Two SNPs (Excalibur_rep_c69981_75 and wsnp_Ra_c2270_4383252) were mapped at 0 cM on the chromosome 6A while one SNP marker (BS00004466_51) was detected at 9 cM. Two regions (Ku_c15750_761 and TA002294-0887) were significantly associated with incubation period on 7A chromosome at 0 cM. The other three markers were significantly associated with incubation period on chromosome 5B (71 and 68 cM) and 6D (117 cM).

Eight SNPs showed significant association with lesion number and were mapped on six chromosomes, 1A, 1B, ID, 5A, 6B and 7B (Table 3). The highest number and most significant (P = 2.62e-08, R^2^ = 0.11) SNP markers for lesion number were on chromosome 6B. The largest numbers of MTAs (-log10; *P*-value>6.0) were on chromosomes 1A, 1B, 5A, 6B, 7Bwhile no MTA was detected on the rest of the chromosomes(Fig 5B).Two SNPs (BobWhite_c17559_105 and wsnp_Ex_c13421_21142520) were mapped within 3 cM on chromosome 1B (P < 1e-06, R^2^ = 0.093). Two SNPs (RAC875_c68978_126 and BS00092845_51) significantly associated with lesion number on 6B chromosome were mapped at 0 cM(P < 1e-06, R^2^ = 0.11), suggesting that one QTL for lesion number was detected by these SNPs (Table 3).The four other single SNPs (RAC875_c32379_216,Excalibur_rep_c105429_528, BobWhite_c23992_300, wsnp_Ex_c2103_3947695) were significantly associated with lesion number on chromosomes 1A, 1D, 5A, 7B at 110, 21, 16 and 50 cM, respectively.

Fourteen SNP markers were found significantly associated with AUDPC and detected on chromosomes 1B, 5B, 6A, 6B(Table 3 and Fig 5C). The most significant associations were recorded for AUDPC on chromosome 5B (P = 7.64e-08, R^2^ = 0.10). The greatest number (11) of SNPs were mapped on chromosome 5B for resistance to spot blotch; of these 11 SNPs, 10were mapped at 0 cM suggesting that one QTL for resistance to spot blotch may underly these SNPs (Table 3).Three other SNPs (BobWhite_c3661_88, BobWhite_c35961_80, Excalibur_c96134_182) were significantly associated with spot blotch AUDPC on different chromosomes (1B, 6A, 6B) (Table 3).

## Discussion

Spot blotch disease of wheat is a major concern in warm humid south Asia and also other countries where similar climatic parameters exist. This is despite the significant progress already made in understanding the biology of *Bipolaris sorokiniana* and also about the cultural practices that can be used to manage this disease. Although some resistance QTL/genes have been identified and resistant cultivars developed, none of these cultivars are completely resistant since resistance is quantitative in nature and controlled by several genes with relatively minor effect. Conventional breeding approaches have contributed immensely to wheat improvement but the progress in developing wheat cultivars resistant to spot blotch has not been satisfactory [29, 30] perhaps because of the complex and polygenic nature of resistance [8, 29].

Initial exploratory analysis of phenotypic data which shows differential performance of each traits across the years, encouraged the environments under investigation is suitable for further analysis. Correlations among the traits are not having much significance suggesting rare common QTL associated with traits under study. Significant differences among genotype and genotype × year observed for all the three traits i.e. incubation period, lesion number and AUDPC revealed the differential performance of all the selected genotypes among themselves as well as across the years for three important traits. The panel exhibited considerable phenotypic, possibly due to the diverse genetic background of the germplasm. Consequently, this result acclaim the selected lines under study are important for further GWAM analysis. In this study, we analyzed the association between spot blotch incubation period, lesion number and AUDPC with SNPs from 287 spring wheat lines in the WAMI panel. We identified 13SNP markers significantly associated with loci conferring resistance to spot blotch AUDPC. In addition, 8 SNPs were identified for incubation period, and 14 for lesion number. Applicability of SNPs for genome-wide association study were verified with already detected DArT markers [31, 32, 33]. GWAM was strengthened by the high-throughput SNP genotyping array and a high-density map to identify putative QTLs associated with spot blotch resistance with better resolution [34].Association mapping is considered superior over bi-parental mapping due to high minor allele frequency, low LD and limited population structure to detect QTLs [32, 35].

Using the WAMI panel in this study, we identified SNP markers linked to five and six QTLs for incubation period and spot blotch lesion number on chromosomes 4A, 6A, 7A, 5B, 6D and 1A, 1B, ID, 5A, 6B, 7B, respectively. Among them, SNPs markers closely linked to one QTL each on the chromosome 4A, 6A and 7A revealed that QTL on the A genome are strongly associated with the incubation period. Likewise, for lesion number, SNPs closely linked to one QTL on the chromosome 6Bwere significant. The associations of incubation period and lesion number with spot blotch resistance has not been reported in previous linkage or GWAM studies.

In addition to incubation period and lesion number, we identified SNP markers linked to four QTLs for spot blotch AUDPC on the chromosomes 1B, 5B, 6A and 6B. Among them, several SNPs closely linked on chromosome 5B. In an earlier association mapping study [32]having an association panel of 566 spring wheat landraces and using 832 Diversity Array Technology (DArT) markers, several genomic regions associated with spot blotch resistance were also identified on four chromosomes (1A, 3B, 7B, 7D). In a follow-up GWAM study, the same group used 528 diverse spring wheat genotypes that were phenotyped for SB and were genotyped using a 9K wheat SNP chip [35], led to the identification of nine associated SNPs located on five different chromosomes (1B, 5A, 5B, 6B, 7B). We detected MTAs that closely corresponded to fourteen loci on four chromosomes that were previously identified and mapped at low resolution by bi-parental QTL analyses using SSRs [22, 29, 30]. The similar position of earlier mapped QTLs with the SNPs of the present study validated QTL for spot blotch resistance.

## Conclusion

SNP markers and GWAS have been useful for QTL discovery in spring wheat. Considerable phenotypic and molecular variation was observed in the WAMI panel used suggesting the diverse genetic background of the germplasm. This study identified 15genomic regions to be associated with resistance to spot blotch disease of wheat. Of these, four regions were for AUDPC, while five and six were for incubation period and lesion number, respectively. A greater number of SNP markers were significantly associated with spot blotch AUDPC than for incubation period and lesion number. This study is the first time association of between markers and incubation period or lesion number were established and QTLs mapped through GWAM. Our data revealed that most of the SNPs were present on the B-genome of wheat. The identified SNP markers linked to resistant QTLs will be useful in augmenting breeding for spot blotch resistance in wheat.

## Supporting information

S1 Table List of wheat association mapping initiative (WAMI) panel of spring wheat along with their phenotypic traits IP (incubation period), LN (lesion number) and AUDPC (Area under disease progress curve) to spot blotch disease. (XLSX)
